# CFD-Based Evaluation of Stirred Tank Designs for High-Viscosity Copolymer Aramid Dope Mixing

**DOI:** 10.3390/polym17233233

**Published:** 2025-12-04

**Authors:** Dong-Hyun Yeo, Hyun-Sung Yoon, Seong-Hun Yu, Jee-Hyun Sim

**Affiliations:** Korea Dyeing & Finishing Technology Institute (DYETEC Institute), Daegu 41706, Republic of Korea; yd@dyetec.or.kr (D.-H.Y.); yoon1216@dyetec.or.kr (H.-S.Y.); enviro1234@dyetec.or.kr (S.-H.Y.)

**Keywords:** computational fluid dynamics (CFD), copolymer aramid dope, mixing performance, stirred tank design, baffle effect

## Abstract

High-viscosity aramid copolymer solutions are widely used in fiber manufacturing and advanced composite applications, but their elevated viscosity poses significant challenges for mixing and agitation processes. This study employs computational fluid dynamics (CFD) simulations to enhance the mixing performance of such systems. Flow behavior around the impeller was analyzed within a cylindrical stirred tank while varying the number of baffles (0, 2, 4, and 6) and comparing two different impeller designs (A and B). Simulation results showed that installing a sufficient number of baffles—particularly four—effectively suppressed swirling flows commonly observed in high-viscosity fluids, thereby significantly improving mixing efficiency. Additionally, impeller geometry played a critical role in performance: the axial-flow impeller promoted faster homogenization and broader circulation compared with the radial-flow design. Through this CFD-based analysis, this study elucidates the key mechanisms governing mixing in high-viscosity fluids and provides practical design and operational guidelines for industrial stirred tank systems. These findings complement existing empirical guidelines focused on low-viscosity fluids and contribute to improving the efficiency and reliability of high-viscosity polymer processing.

## 1. Introduction

Aramid fibers are critical materials in defense, aerospace, and industrial textiles owing to their exceptional thermal and mechanical properties [1,2]. Among them, copolymer aramids offer improved solubility and processability compared with para-aramids, making them favorable for wet-spinning processes [3,4]. These materials are typically synthesized via solution polycondensation and used directly as spinning dopes, where a uniform molecular weight distribution is essential to ensure consistent fiber quality [2,5]. Achieving such uniformity demands efficient mixing of monomers and intermediates throughout polymerization, emphasizing the importance of optimized agitation in the reactor [6].

However, mixing highly viscous aramid copolymer dopes poses substantial challenges. The flow tends to localize near the impeller, forming extensive dead zones and leading to nonuniform mixing and incomplete reactions, which ultimately degrade polymer properties [7,8]. The high viscous resistance not only increases torque demand and energy consumption but also raises the risk of mechanical failure in the impeller system [9]. Therefore, enhancing mixing performance under high-viscosity conditions is a key concern for scale-up and production stability [10].

The use of baffles is a common strategy to suppress swirling and enhance axial and radial flow patterns, promoting better dispersion and reducing stagnation zones [11,12]. Nonetheless, their effectiveness is highly dependent on fluid rheology, impeller geometry, and operational parameters. Overuse of baffles may increase hydrodynamic drag and energy load without proportionate mixing benefits [13]. Despite their importance, systematic investigations into baffle-assisted mixing for high-viscosity copolymer systems—especially at pilot or industrial scale—remain limited. Experimental studies are often costly and difficult to generalize due to scale-specific turbulence behavior [14].

Beyond fiber production, high-viscosity copolymer systems are also extensively applied in other fields such as enhanced oil recovery (EOR), where viscoelastic polymer flooding improves sweep efficiency and displacement performance. The interplay of elasticity and wettability in such applications has been highlighted in recent studies [15], further underscoring the need for robust mixing control in high-viscosity polymer handling.

To characterize mixing regimes, the Reynolds number (Re)—defined as the ratio of inertial to viscous forces—is frequently used. It distinguishes laminar (Re < 2 × 10^3^), transitional (2 × 10^3^ ≤ Re < 4 × 10^3^), and turbulent (Re > 4 × 10^3^) flow regimes, with turbulence being generally favorable for uniform mixing and energy transfer [16,17]. In the context of high-viscosity agitation, Re is a critical design parameter.

Accordingly, this study utilizes computational fluid dynamics (CFD) to analyze the mixing behavior of a highly viscous aramid copolymer dope in a stirred tank reactor equipped with an anchor-type impeller. A parametric study was conducted to evaluate the influence of baffle number (0, 2, 4, 6) on flow characteristics such as velocity fields, turbulence intensity, and mixing homogeneity. Furthermore, two mixing systems were compared to assess their impact on circulation and efficiency. The goal was to identify agitation designs and operational conditions that ensure mechanical stability while maximizing mixing effectiveness. This study addresses a critical knowledge gap in the field and offers practical design insights for industrial-scale processing of viscous polymer systems [18,19].

## 2. Theoretical Background and Numerical Methods

### 2.1. Governing Equations and Turbulence Model

The flow was modeled as three-dimensional, steady-state, single-phase, incompressible turbulent flow [20]. The governing equations were the continuity and Navier–Stokes momentum equations [21,22]:

Continuity: ∇·u = 0(1)

Momentum: ρ(∂u/∂t + (u·∇)u) = −∇p + ∇·(τ + τ_R_) + ρg, (2)

with τ = μ[∇u + (∇u)^T^] for Newtonian fluids. The Reynolds stress τ_R_ was modeled via the standard k–ε model using Boussinesq’s hypothesis, with transport equations for k and ε and model constants σ_k_, σ_ε_, C_ε1_, and C_ε2_ [23,24].

### 2.2. Numerical Methodology

**Figure 1:**
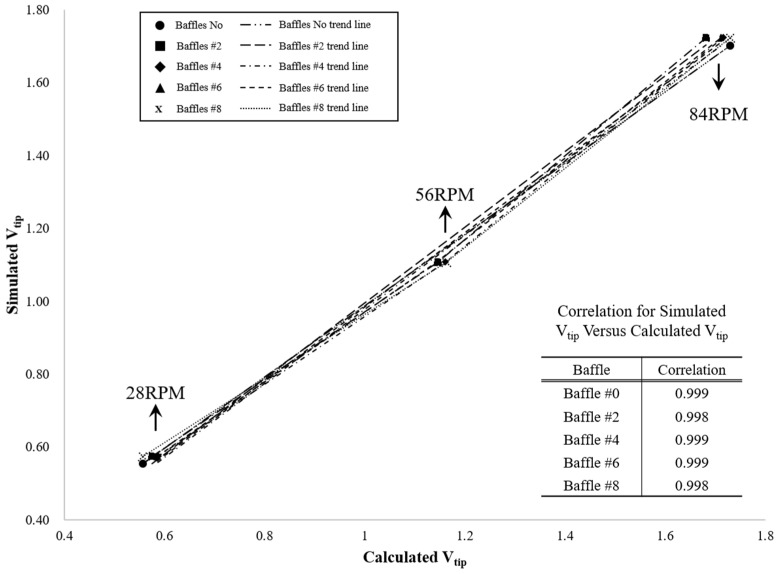
Correlation between calculated and simulated tip speeds (V_tip_) of the anchor impeller under different baffle configurations.

CFD was performed using SolidWorks Flow Simulation 2023 (Dassault Systèmes, Vélizy-Villacoublay, France) with a finite-volume formulation [25]. Least-squares cell-based gradients and first-order upwind for convection were used; pressure–velocity coupling employed SIMPLE [21]. The rotating dual-impeller system was modeled using MRF: a rotating frame around the shaft (upper pitched paddle and lower anchor) and a stationary frame for the tank/baffles, exchanging variables across the interface [26]. No-slip conditions were applied on walls, baffles, and impellers; impeller speed was imposed in the MRF region. An unstructured mesh (locally refined near impellers/walls) ensured accuracy; convergence was determined by residuals (e.g., <10^−4^) and stabilization of monitored quantities such as impeller torque [25]. According to Figure 1, the reliability of the model was verified by comparing the theoretical tip speed [26]:

V_tip_ = πD_anchor_RPM/60 (with D_anchor_ ≈ 0.94 m) (3)

with the simulated tip velocities. The close agreement observed across all baffle configurations confirms the fidelity of the CFD model.

## 3. Target System and Condition

### 3.1. Copolymer Aramid Solution and Properties

**Figure 2:**
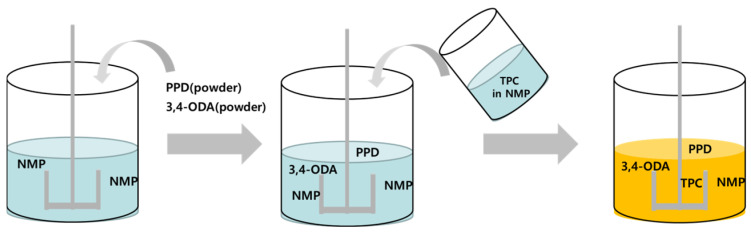
Schematic illustration of the copolymer aramid dope preparation process: dissolution of PDA and 3,4′-ODA in NMP, followed by addition of TPC in NMP.

As illustrated in Figure 2, the copolymer aramid dope was prepared by dissolving p-phenylenediamine (PDA) and 3,4′-oxydianiline (ODA) in N-methyl-2-pyrrolidone (NMP), followed by the addition of terephthaloyl chloride (TPC) to initiate solution polycondensation [1,2,3]. The resulting dope contained 6.0 wt% copolymer aramid at 80 °C, with a volume of 0.5 m^3^ and a density of 1.12 g/cm^3^ [2,5]. To capture the evolution of viscosity during polymerization, both an initial low-viscosity state (~1.2 cP at 80 °C) and a final high-viscosity state (1.5 × 10^5^–2.5 × 10^5^ cP at 80 °C) were considered in the analysis [2].

### 3.2. Stirred Tank and Impeller Geometry

The reactor is cylindrical (Inner Diameter 0.94 m) with a dished bottom, a straight-side height of 1.1743 m, and a bottom depth of 0.235 m, and was supplied by Iljin A-Tech (Ulsan, Republic of Korea). The working volume corresponds to ~0.9 m^3^ [6]. As shown in Table 1, the dual-impeller configuration comprises an upper 4-pitched paddle (Ø ~0.5 m) to promote axial flow and vertical mixing and a lower two-blade anchor (blade height 0.5 m, Ø ~0.9 m) to induce radial/tangential flow near the wall for high-viscosity mixing and heat transfer [7,8,9,10]. The system is driven by an 11 kW three-phase induction motor; wetted parts are SUS316L [11].

### 3.3. Baffle Configuration and Parametric Study

As shown in Table 2, the computational domain and mesh configuration were constructed for stirred tanks with and without baffles. The baseline model included two vertical baffles (width 0.094 m) positioned 180° apart on the tank wall. Four different configurations—0, 2, 4, and 6 baffles—were modeled to evaluate their effects on flow structure, circulation patterns, and overall mixing characteristics [8,12,13]. The mesh was generated using a hexahedral-dominant hybrid scheme with local tetrahedral refinement near the curved boundaries. The total number of elements was approximately 1.2 × 10^6^, with a minimum cell size of 1.0 mm near the impeller tip and wall region, and a maximum of 10 mm in the bulk zone. Inflation layers (5–7 layers, growth rate 1.2) were applied along the wall to accurately capture near-wall velocity gradients. Grid-independence tests were performed at 0.8 × 10^6^, 1.2 × 10^6^, and 1.6 × 10^6^ elements, confirming numerical stability at around 1.2 × 10^6^ cells.

### 3.4. Simulation Conditions and Properties

Single-phase steady simulations were conducted [16,17]. Properties and settings:

- Working fluid: NMP-based copolymer aramid dope;
- Density: 1.12 g/cm^3^ at 80 °C;
- Viscosity set: 1 cP (NMP), 5.0 × 10^4^, 1.0 × 10^5^ cP, 1.5 × 10^5^, 2.0 × 10^5^, and 2.5 × 10^5^ cP;
- Geometry: tank Ø 1.0 m, H 1.2 m; upper 4-pitched paddle Ø 0.5 m; lower two-anchor Ø ~0.9 m, blade height 0.5 m; baffles = 0/2/4/6, cross-section h 0.09 m × L 1.2 m;
- Operating: 80 °C; impeller speeds in a representative range ~28–84 RPM subject to motor/viscosity limits;
- Analysis metrics: Reynolds number; velocity fields; k–ε distributions; impeller torque/power; pressure; qualitative mixing uniformity [19,20,21,26].

## 4. Results

### 4.1. Effect of Fluid Viscosity

**Figure 3:**
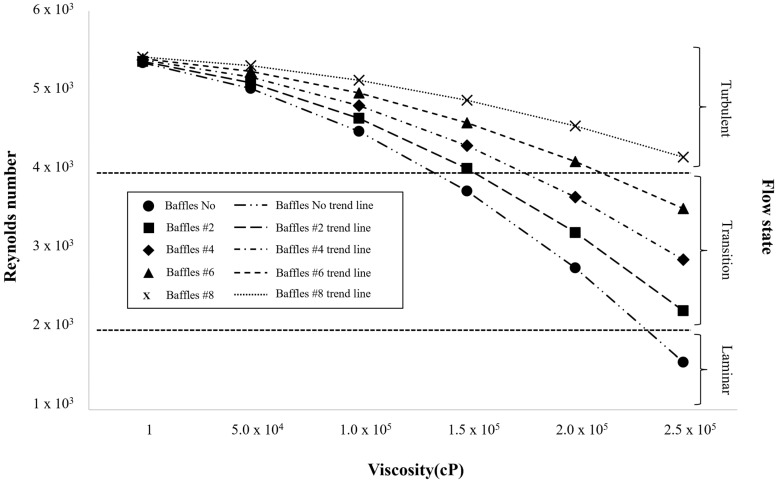
Variation in Reynolds number with viscosity for different baffle configurations (0, 2, 4, 6, and 8), indicating the transition boundaries between turbulent, transitional, and laminar flow regimes.

As shown in Figure 3, the Reynolds number decreased progressively with increasing viscosity across all baffle configurations (0, 2, 4, 6, and 8), reflecting the dominance of viscous forces over inertial effects. At low viscosities, inertial transport was sufficient to sustain turbulent eddies (Re > 4 × 10^3^), allowing vigorous momentum exchange and efficient mixing throughout the vessel. However, as viscosity increased, momentum diffusion intensified, damping the development of turbulent vortices and reducing flow instability. At around 1.5 × 10^5^ cP, the system entered the transitional regime as viscous dissipation and began to exceed inertial energy input from the impeller.

In this intermediate range, the hydrodynamic role of baffles became particularly important. Additional baffles disrupted the tangential swirling motion and redistributed kinetic energy into axial and radial directions, helping maintain slightly higher Reynolds numbers and improve circulation near the impeller. Yet, when viscosity exceeded approximately 1.8 × 10^5^ cP, viscous stresses became the primary governing force, suppressing large-scale vortex structures and confining motion to narrow shear layers adjacent to the impeller blades. At 2.5 × 10^5^ cP, the flow approached a quasi-laminar state, in which energy from the impeller was rapidly dissipated near the wall, and core stagnation zones expanded significantly. These observations confirm that viscosity dictates the overall flow regime by controlling the balance between inertial and viscous forces, while the geometric influence of baffles becomes secondary once the system transitions into a viscosity-dominated regime.

### 4.2. Spatial Flow Distribution

**Figure 4:**
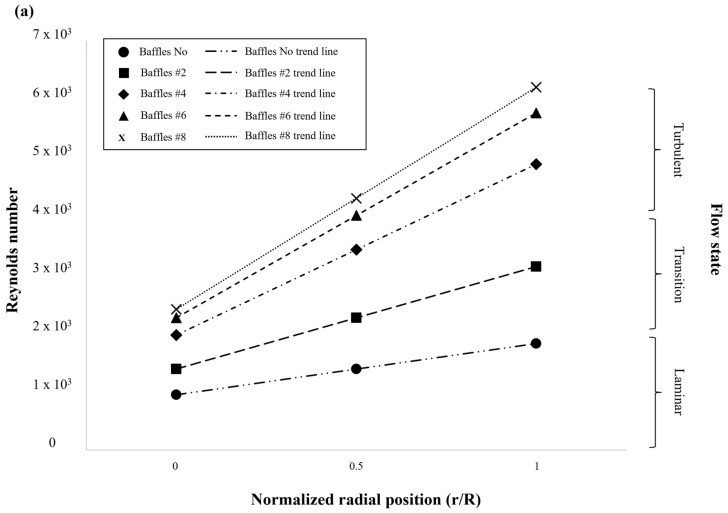

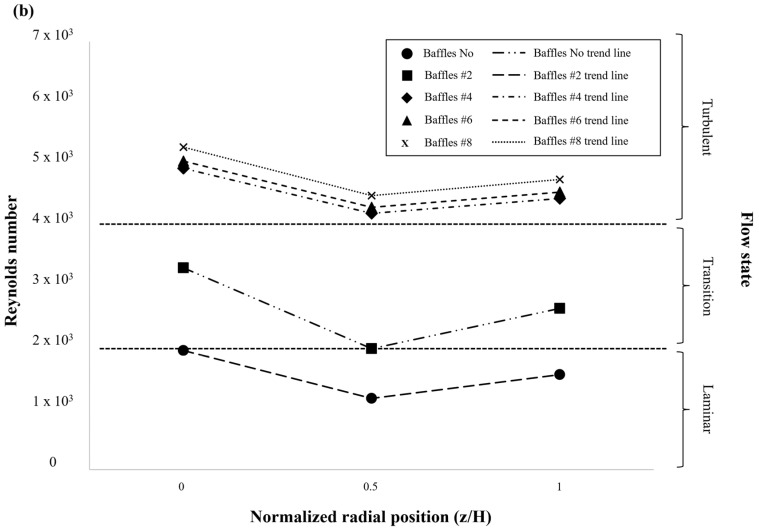
Radial and axial distributions of Reynolds number (Re) in the stirred tank for different baffle configurations. (**a**) Radial variation across the normalized tank radius (r/R), showing increasing Re toward the wall due to enhanced impeller–baffle interactions and wall shear effects. (**b**) Axial variation along the normalized depth (z/H), indicating reduced circulation and lower Re near the upper region, where viscous damping dominates and flow transitions toward the laminar regime.

As shown in Figure 4, the Reynolds number distribution exhibited distinct nonuniformity in both the radial (a) and axial (b) directions, demonstrating the interplay between impeller-induced flow, viscous resistance, and baffle-induced shear layers within the high-viscosity system.

In the radial direction (Figure 4a), the Reynolds number increased progressively from the tank centerline (r/R ≈ 0) toward the wall (r/R = 1). Near the centerline, the influence of the impeller was minimal due to the small local rotation radius and weak inertial transport, resulting in a viscous-dominated core region characterized by low flow energy and poor mixing. Moving outward, impeller–baffle interactions intensified, producing strong shear layers and secondary vortices along the tank wall. These effects redistributed tangential momentum into radial components, forming outward jets that enhanced local mixing and wall-shear-driven heat transfer. The presence of multiple baffles further strengthened these interactions by suppressing circumferential swirling and promoting momentum coupling between adjacent flow cells, thereby improving flow uniformity near the wall region.

In the axial direction (Figure 4b), the Reynolds number generally decreased from the impeller zone toward the upper free surface, highlighting the limited vertical pumping ability of the anchor impeller. The upper region was dominated by viscous diffusion, where the attenuation of large-scale vortical structures led to the formation of quasi-stagnant zones. Under moderate viscosity conditions (e.g., 5 × 10^4^ cP), a continuous circulation loop was sustained throughout the vessel. However, as viscosity increased to 2.5 × 10^5^ cP, the dominance of viscous stresses suppressed upward flow propagation, resulting in stratified layers and stagnation pockets in the upper domain.

Overall, these results demonstrate that spatial nonuniformities in high-viscosity mixing originate from the imbalance between inertial momentum and viscous dissipation. Baffles mitigate this imbalance by generating strong shear coupling between tangential and radial directions, thereby stabilizing circulation and enhancing mixing efficiency. Nevertheless, as the system transitions into a viscosity-dominated regime, the impeller’s input energy is rapidly dissipated near the blades and walls, reducing its capacity to sustain large-scale flow circulation across the tank.

### 4.3. Effect of Baffle Number

**Figure 5:**
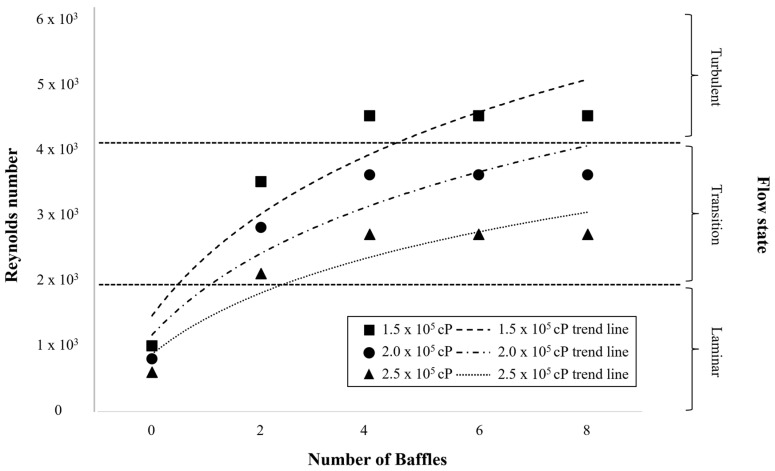
Variation in Reynolds number with baffle number at viscosities of 1.5 × 10^5^, 2.0 × 10^5^, and 2.5 × 10^5^ cP, showing the effect of baffle addition on flow transition and mixing performance.

As shown in Figure 5, the Reynolds number increased with the number of baffles for all tested viscosities (1.5 × 10^5^, 2.0 × 10^5^, and 2.5 × 10^5^ cP), reflecting the enhancement of momentum exchange and secondary circulation induced by baffle-generated shear layers. The addition of baffles disrupted the circumferential vortex formed by the impeller and redirected kinetic energy into axial and radial components, thereby promoting more efficient momentum transfer throughout the tank. At 1.5 × 10^5^ cP, two or more baffles were sufficient to break the rotational symmetry and generate stable radial jets, enabling a transition to turbulence (Re > 4.0 × 10^3^). This finding indicates that under moderate viscosity, baffles effectively convert impeller-driven tangential momentum into three-dimensional circulation that sustains turbulent energy dissipation.

At 2.0 × 10^5^ cP, viscous damping became dominant, reducing inertial transport and limiting the penetration depth of impeller-induced flow. Although additional baffles increased local shear intensity near the wall, the bulk flow remained in the transitional regime (Re ≈ 3.0 × 10^3^–3.5 × 10^3^), suggesting that viscous diffusion suppressed large-scale vortical motion. At 2.5 × 10^5^ cP, the flow was almost entirely governed by viscous forces; the Reynolds number approached ~2.0 × 10^3^, and energy input from the impeller was rapidly dissipated near the blade tips, confining motion to narrow shear bands. Consequently, although baffles are essential for inducing turbulence and enhancing mixing at moderate viscosity, their effect diminishes once the viscous stresses dominate over inertial forces, establishing a viscosity-controlled flow regime.

### 4.4. Operational Constraints and Optimization

**Figure 6:**
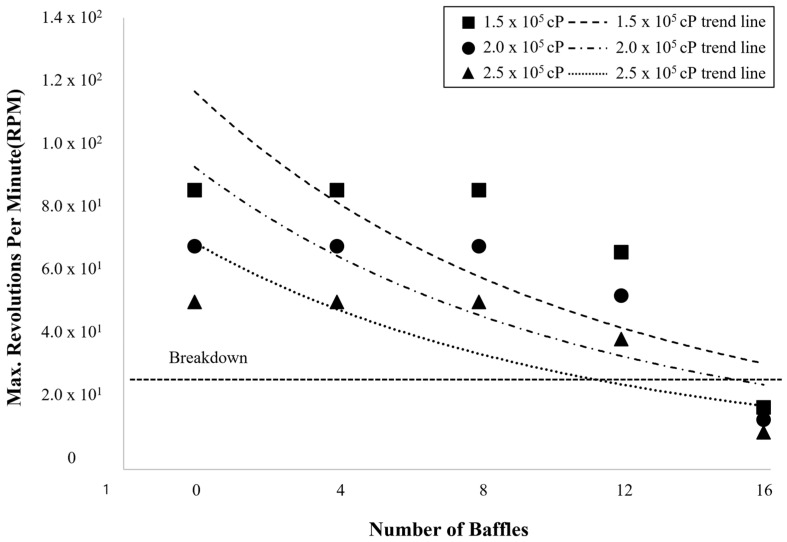
Maximum allowable impeller speed as a function of baffle number at viscosities of 1.5 × 10^5^, 2.0 × 10^5^, and 2.5 × 10^5^ cP, showing decreasing RPM with increasing viscosity and baffles, and the approach to the breakdown region (≤26 RPM).

As shown in Figure 6, the maximum allowable impeller speed decreased with both increasing viscosity and baffle number due to enhanced hydrodynamic resistance and torque load on the motor. Physically, the rise in viscosity increases the momentum diffusion within the fluid, amplifying viscous shear stress on the impeller surface. Simultaneously, additional baffles disrupt the circumferential flow and create secondary shear layers along the wall, which further elevate the overall drag torque acting on the shaft.

At moderate viscosity (1.5 × 10^5^cP), the impeller could rotate at higher speeds because the inertial effects remained sufficient to overcome viscous damping, maintaining stable circulation. However, as viscosity increased to 2.0 × 10^5^–2.5 × 10^5^ cP, viscous stresses dominated, and the fluid exhibited a strong resistance to deformation. Under these conditions, torque demand rose nonlinearly, causing a rapid decline in allowable RPM—from about 50 RPM without baffles to nearly 12 RPM with multiple baffles—approaching the mechanical breakdown threshold (≤26 RPM).

This trend highlights the coupled interaction between viscous dissipation and mechanical load: higher viscosity increases the energy required to generate flow, while more baffles enhance localized shear zones, which contribute to torque buildup. Consequently, in high-viscosity regimes, maintaining stable agitation requires careful balancing between flow generation and motor capacity. Reducing fluid viscosity (e.g., by temperature control), limiting baffle count to 2–4, or employing impeller geometries with larger flow channels (such as helical ribbon or RCI designs) can effectively mitigate excessive torque and ensure operational stability.

### 4.5. Impact on Final Product Properties

As shown in Table 3, to link hydrodynamics to material outcomes, we compared System A (conventional) with System B (Improved), which was designed to minimize dead zones and enhance uniformity. The dope viscosity, number-average molecular weight (Mn), and drawn-fiber tensile strength improved markedly with Agitator B: viscosity 140→300 Pa·s, Mn 223,700→368,000 g mol^−1^, tensile 18.5→21.4 g/d. Interpretation: (1) better mixing increased monomer contact and reaction completion, yielding higher Mn; (2) higher Mn accounts for the viscosity rise; (3) higher Mn and improved molecular entanglement/orientation during spinning enhanced tensile properties.

## 5. Conclusions

This study employed CFD simulations to elucidate the hydrodynamic mechanisms governing the mixing of highly viscous copolymer aramid dopes in a stirred tank equipped with an anchor impeller under various baffle configurations. The analysis revealed that viscosity is the dominant factor controlling the flow regime, as it determines the balance between inertial transport and viscous dissipation within the reactor. Increasing viscosity suppressed large-scale turbulence and promoted the formation of confined shear layers near the impeller, leading to localized mixing and extensive stagnation zones at high viscosities (>1.8 × 10^5^ cP).

From a fluid dynamic standpoint, baffles were shown to convert tangential momentum into axial and radial circulations through shear-layer generation and flow redirection. This mechanism enhanced turbulence and mixing uniformity at moderate viscosities (~1.5 × 10^5^ cP). However, beyond a critical viscosity threshold, viscous damping overwhelmed inertial forces, reducing the efficacy of additional baffles and establishing a viscosity-dominated regime in which geometric optimization yields diminishing returns. The coupling between viscous energy dissipation and mechanical torque load further constrained operational limits, as the allowable impeller speed decreased nonlinearly with both viscosity and baffle number.

These findings provide quantitative engineering insights for designing and scaling high-viscosity polymer mixing systems. Practically, maintaining the fluid viscosity below ~1.5 × 10^5^ cP, limiting baffles to 2–4, and selecting impeller geometries that favor large-scale axial circulation (e.g., helical ribbon or RCI designs) can ensure both mixing efficiency and mechanical stability. Importantly, improved hydrodynamics directly translated into enhanced material properties—such as higher molecular weight and superior tensile strength—demonstrating the strong linkage between reactor fluid mechanics and polymer performance.

Overall, this study establishes a mechanistic and application-oriented framework for optimizing high-viscosity mixing processes. The CFD-based approach not only clarifies the interplay between flow physics, geometry, and material outcomes but also offers a scalable design strategy applicable to industrial polymerization and spinning operations requiring precise control of mixing, viscosity, and product uniformity.

## Figures and Tables

**Table 1:** Geometrical representation of the stirred tank and impeller system. The geometry column illustrates the overall 3D configuration, the section view highlights the internal structure, and the projection view shows the orthographic representation used for computational modeling. Arrows are incorporated to clearly indicate the impeller rotation direction and the corresponding flow orientation within the reactor.

Geometry	Section View	Projection View
Impeller	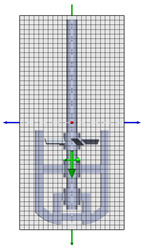	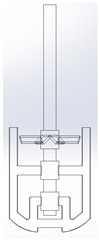
Tank	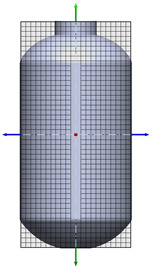	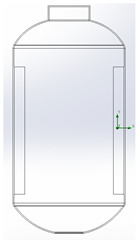

**Table 2:** Geometrical configurations of the stirred tank with different baffle numbers: (a) no baffle; (b) two baffles (180° interval); (c) four baffles (90° interval); (d) six baffles (60° interval); (e) eight baffles (45° interval).

Baffle Number	Top View	Isometric View
(a) Baffle = 0	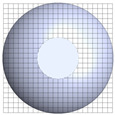	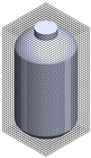
(b) Baffle = 2	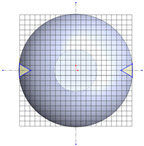	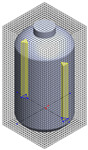
(c) Baffle = 4	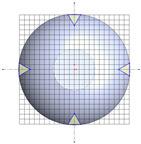	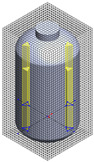
(d) Baffle = 6	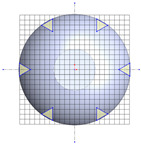	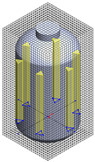
(e) Baffle = 8	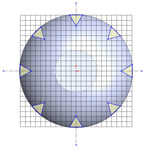	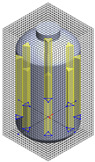

**Table 3:** Comparison of the conventional and improved mixing systems: (a) Conventional mixing system without baffles (Baffle = 0) and (b) improved mixing system with four baffles (Baffle = 4). The isometric views illustrate the geometric configurations, while the CFD velocity vector fields and flow trajectories visualize the internal flow structures within the stirred tank, highlighting enhanced circulation and reduced stagnation in the baffled system.

Baffle Number	Isometric View	Velocity Vector Field and Flow Trajectories Inside the Stirred Tank
(a) System A (Conventional) (Baffle = 0)	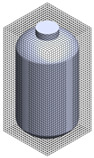	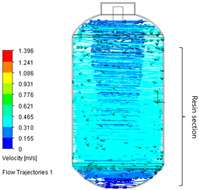
(b) System B (Improved) (Baffle = 4)	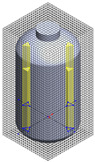	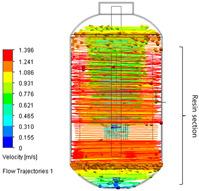

## Data Availability

The data presented in this study are available from the corresponding author upon reasonable request. The data are not publicly available due to institutional restrictions.

## Abbreviations

The following abbreviations are used in this manuscript:

CFD	Computational fluid dynamics
MRF	Multiple reference frame
Re	Reynolds number
NMP	N-Methyl-2-pyrrolidone
PDA	p-Phenylenediamine
ODA	3,4′-Oxydianiline
TPC	Terephthaloyl chloride
Mn	Number-average molecular weight
RPM	Revolutions Per Minute
Pa·s	Pascal second (unit of dynamic viscosity)
k–ε model	Turbulence model based on turbulent kinetic energy (k) and dissipation rate (ε)
SIMPLE	Semi-implicit method for pressure-linked equations
